# Mind the Global Community Health Funding Gap

**DOI:** 10.9745/GHSP-D-20-00517

**Published:** 2021-03-15

**Authors:** Angela Gichaga, Lizah Masis, Amit Chandra, Dan Palazuelos, Nelly Wakaba

**Affiliations:** a Financing Alliance for Health.; b Harvard Medical School, Boston, MA, USA.; c Partners In Health, Boston, MA, USA.

## Abstract

Community health workers play a critical role in providing both essential health services and pandemic response. Community health demonstrates a strong return on investment, but funding for this sector is limited and fragmented. Understanding the underlying costs of a community health system is crucial for both planning and policy; the data demonstrate a strong investment case.

## Key Messages

- Community health workers (CHWs) play a critical role in bridging the huge human resources for health gap while providing both essential health services and pandemic response.
- The case for investment is strong but funding for this sector is limited and fragmented. The current funding gap for at scale CHW program in sub-Saharan Africa has increased from an estimated US$3.1 billion to US$5.4 billion annually but is still cost effective as the cost per capita remains low (US$1.50–US$13.00).
- To close this funding gap, political prioritization, supportive policies, programs designed to deliver, and reducing fragmentation of existing resources will be critical.
- Governments can achieve universal health coverage targets by building community health systems that are people centered and quality oriented.
- Funders should also take a systems approach and focus their investments toward building resilient and sustainable platforms of delivery.

## COMMUNITY HEALTH SYSTEMS ARE CRITICAL

The value of community health workers (CHWs) cannot be overstated: they provide basic health care and health promotion within the communities they live.^1^ In sub-Saharan Africa (SSA), which has a gross shortage of health care workers of only 5 health professionals per 10,000 population—compared to a global threshold of 23 per 10,000— CHWs are part of the solution through task sharing^2^ and an increase in scope of work.^1^ Aditionally, during the recent Ebola virus disease outbreak in West Africa and the global coronavirus disease (COVID-19) pandemic, CHWs played a critical role in mobilizing communities, finding active cases, and filling health service gaps.^3^

Existing evidence posits that CHW programs are a high-value investment in health as the annual cost per capita served is comparatively low.^1^ Additionally, expanded access to key interventions by CHWs could avert up to 3 million deaths annually.^4^ One analysis demonstrated a 10:1 economic return on investment from community health systems in SSA.^4^ This return on investment is attained through CHWs' effects on increasing productivity from a healthier population, contributions to effective prevention which circumvents costly health crises, and the economic gains from increased employment, particularly for women who may make up to 70% of the community health workforce.^5^

Some analysts question whether CHW programs are affordable in the most impoverished countries and whether CHW program ambitions should be scaled back.^1^ This article maps a series of pathways, tested in countries by the Financing Alliance for Health and its partners, that even the most under-resourced governments can follow to avoid abandoning opportunities to improve the health of their communities and grow their economies. Every country should have a plan for their health system. Even starting with realistic ambitions and high affordability starts the process of prioritizing health and offers a point from which to grow.

We map a series of pathways that even the most under-resourced governments can follow to avoid abandoning opportunities to improve the health of their communities and grow their economies.

## DESPITE THE STRONG CASE FOR INVESTMENT, COMMUNITY HEALTH SYSTEMS ARE UNDERFUNDED

**FIGURE 1:**
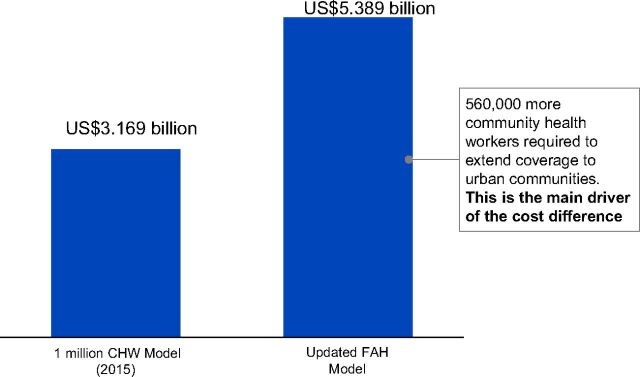
Updated Annual Total Costs of At-Scale Community Health Worker Program Resource Needs in Sub-Saharan Africa,^a^ by Model US$Billions Abbreviations: CHW, community health worker; FAH, Financing Alliance for Health.^a^ Key driving factors of cost are rural versus rural and urban coverage (62% versus 100% of sub-Saharan Africa population) and higher cost per community health worker (11% difference).

**FIGURE 2:**
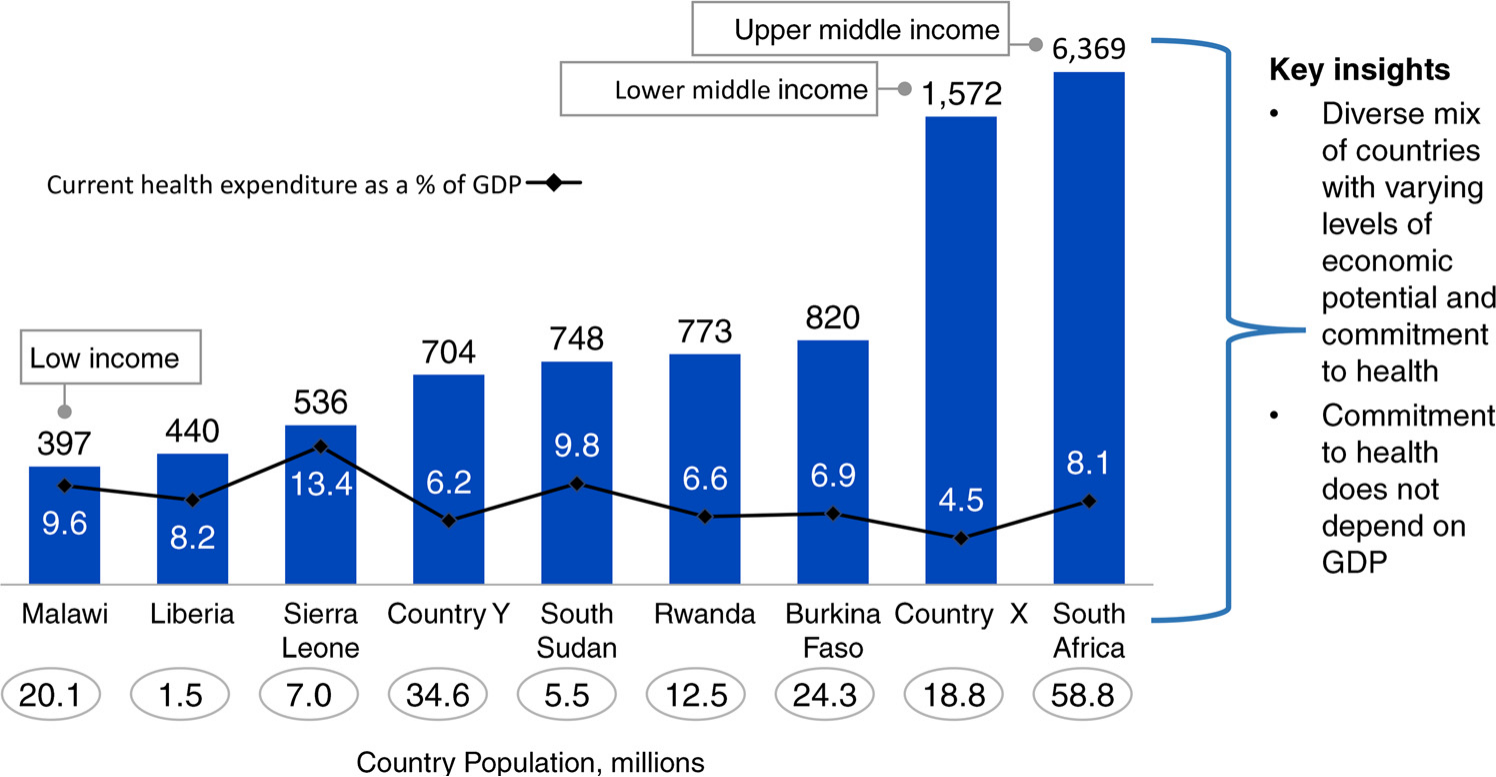
Cost Comparison of National Community Health Programs Across 9 Countries^a^ in Sub-Saharan Africa, GDP Per Capita, US$ Abbreviation: GDP, gross domestic product.^a^ Countries X and Y are masked - awaiting formal government approval to share the data.

The community health financing landscape has changed immensely since we published a report in 2017 on closing the US$2 billion gap^6^; more countries have begun their journey toward universal health coverage by launching ambitious countrywide community health programs and generating a wealth of primary programmatic data.^6^ As a result, following a comprehensive comparison of costs across national programs based on the available country data, the annual resource needs for at-scale community health systems in SSA has increased from US$3.1 billion to US$5.4 billion (Figure 1). The main driver of this cost is the increased coverage to include both rural and urban population.^7^ This cost comparison was considered across 9 countries (Figure 2).

**FIGURE 3:**
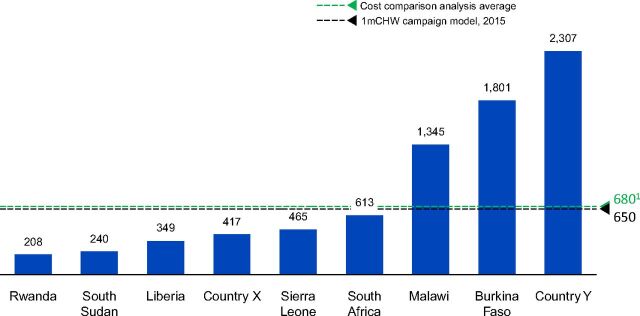
Average Community Health Worker to Population Ratio in 9 Countries in Sub-Saharan Africa ^a^ Excludes country Y in the average number; model only costed one cadre who play more of a supervisory role and that serve entire population but did not include volunteer CHWs because the program has not been costed. Costs are based on recurrent costs including commodities. Costs reflected are final year costs for the duration of the community health strategy (and hence final year of costing model). This assumes that at the final year, the program will be fully scaled, hence will have reached the steady state. Steady state costing values are adjusted to 2019 US$for comparison. Countries X and Y are masked - awaiting formal government approval to share the data.

Consequently, the average CHW to population coverage ratio in SSA was 1:680 and varied widely from ∼200–2,400 people per CHW.^7^ This implies that approximately 1.6 million CHWs are needed in SSA and a gap of more than 600,000 CHWs currently exists, assuming that the 1 million campaign was on target in 2015 (Figure 3). However, given that by September 2016, the available data from 37 countries in SSA showed that only 332,000 CHWs of the 1 million CHWs campaign existed^1^; this would translate to a CHW gap of about 1.3 million.

**FIGURE 4:**
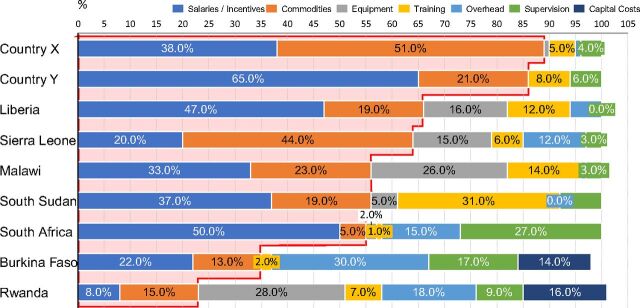
Community Health Annualized Program Costs Showing Salaries and Incentives as the Main Cost Drivers Across 9 Countries in Sub-Saharan Africa^a,b^ ^a^ Commodities and salaries/incentives were the main cost drivers accounting for between 50%–90% of costs. Countries X and Y are masked; awaiting formal government approval to share the data.^b^ Key insights: overhead costs were relatively higher for countries with whole directorates; inclusion of mobile phones resulted in relatively higher costs; countries with longer training programs had relatively higher training costs.

**FIGURE 5:**
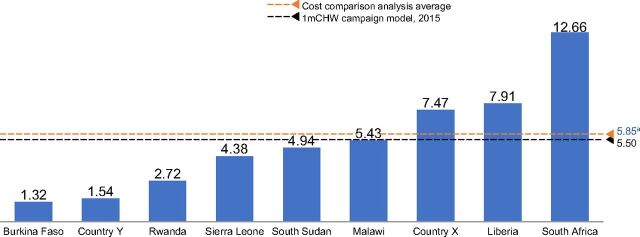
The Average Annual Cost per Capita Served Across 9 Countries in Sub-Saharan Africa, US$^a^ ^a^ The annual average cost per person served ranged from $1.50–$13.0. Excludes country Y in the average number; model only costed one cadre who play more of a supervisory role and that serve entire population but did not include volunteer CHWS because the program has not been costed; costs are based on recurrent costs including commodities; costs reflected are final year costs for the duration of the community health strategy (and hence final year of costing model). This assumes that at the final year, the program will be fully scaled, hence will have reached the steady state. Steady state costing values are adjusted to 2019 USDs for comparison. Countries X and Y are masked; awaiting formal government approval to share the data.

**FIGURE 6:**
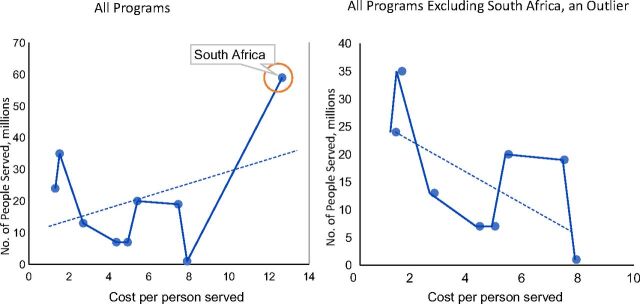
Cost Efficiency of Community Health Programs With Scale in 9 Countries in Sub-Saharan Africa^a^ ^a^ As the number of people served increases, cost per person served decreases. South Africa is an outlier with both a higher population and higher cost structure for its community health program, which is based on high-cost ward-based outreach teams, a multidisciplinary platform integrated into primary care. Excluding South Africa, the trendline goes downward indicating economies of scale likely due to shared fixed costs.

Despite the high total costs^1^ (unpublished data), this analysis demonstrated that community health programs at scale offer considerable value for the investments made (Figure 4); annual average cost per person served ranged from US$1.50–US$13.00 (Figure 5) and, as number of people served increases, cost per person served decreases, indicating greater cost efficiency with scale (unpublished data) (Figure 6). In comparison, a primary health care (PHC) system in low-income countries would cost between US$50–US$55 per capita per year.^7^

There are 4 main reasons why this gap in funding exists based on the available evidence as well as our experience working with governments on community health in SSA. We highlight these and propose solutions that could be a pathway to closing this funding gap.

### 1. Lack of Political Prioritization

Beyond the prevailing economic situation in a country, political prioritization greatly influences the allocation of resources. Diverse champions for community health are needed to advocate for support and increased funding. Juxtaposed in this is a myriad of competing health priorities^1^ and an ever-shrinking fiscal space. Maximizing on existing windows of opportunity, such as a budgeting process or favorable political climate, and providing evidence that supports the investment in community health to the influential champions would influence the allocation of resources needed in closing this funding gap.^4^

For example, in Ethiopia, strong political will in 2000s from then Prime Minister Meles Zenawi and then Minister of Health Tedros Adhanom Ghebreyesus ensured buy-in for the Health Extension Program of Ethiopia at all levels of government, including multisectoral collaboration. With approximately 40,000 health extension workers and about 4 million health development army volunteers, the program has greatly contributed to the more than doubling of immunization rate and contraceptive prevalence while also increasing skilled birth attendance by up to 10 times.^4^^,^^8^^–^^11^ This high political prioritization resulted in required financing from domestic resources as well as pooled and earmarked funding from donors—usually an uphill task given the rampant vertical and silo funding. However, in 2006–2010, the health extension program received more than US$180 million in pooled funding (Table).^12^^–^^14^

**TABLE.** Funding Flows for the Health Extension Program in Ethiopia

	Channel 1: Ministry of Finance	Channel 2: Ministry of Health	Channel 3: Outside of GOE Oversight
Description	Flows via Ministry of Finance. Includes unearmarked general budget support from donors (PBS) and GOE, and program-specific funds from some donors	Flows via Ministry of Health. Includes pooled donor funds (M/SDG fund) and program-specific funds from some donors	Flows from donors via implementing partners, largely outside of GOE oversight (but aligned with government strategies)
% of total health fundinga	50% (includes Government of Ethiopia and donor budget support funds)	25%	25%
Key mechanisms	**PBS:** Pooled donor fund launched in 2006 to provide general budget support for basic services (across sectors) via federal block grants. ∼20% of PBS at woreda level used in health, largely for HEW salaries, and some for procurement	**M/SDGPF:** Non-earmarked pooled donor fund for health sector support, launched in 2008. Scope of activities determined through consultative process and joint financing agreement each year. Funds supplies, training, construction (not salaries). Became SDGPF in 2015.	
Major donor contributors	PBS: CIDA, Italy, Netherlands, World Bank Other Channel 1: Austria, Spain, Irish Aid, UNICEF, UNFPA, WHO	M/SDGPF: DFID, Irish Aid, Italy, Spain, Netherlands, Gavi, UNFPA, WHO, World Bank Other Channel 2: UNDP, CIDA, Italy, USAID, World Bank, Global Fund	USAID, PEPFAR, CDC (largest) Most other bilateral and some multilateral donors provide some funds through channel 3

Abbreviations: CDC, U.S. Centers for Disease Control and Prevention; CIDA; Canadian International Development Agency; DFID, United Kingdom Department for International Development; GOE, Government of Ethiopia; M/SGDPF, Millennium/Sustainable Development Goal Performance Fund; PBS, Promoting Basic Services Program; PEPFAR, U.S. President's Emergency Plan for AIDS Relief; UNDP, United Nations Development Programme; UNFPA, United Nations Population Fund; UNICEF, United Nations Children's Fund; USAID, U.S. Agency for International Development; WHO, World Health Organization.

a Approximated based on Harvard/Ministry of Health data (2010).^14^ Estimates of % of funding through each channel are order of magnitude based on Harvard/Ministry of Health data from 2010. Indicative, not comprehensive.

### 2. Lack of Supportive Policies, Strategies, and Investment Case Documents

It is critical to also document this political will in the form of a community health policy and a community health strategy, both of which should be linked to the overall national health policies and strategic development plans. Evidence shows that the effectiveness of CHW systems also depends on how well the rest of the health system functions because CHW programs are not “stand alone.” Hence, other levels of care, such as the PHC level, provide logistical support, supervision support, and adequate supplies for CHWs. Therefore, it is imperative that CHW programs are embedded within a functional PHC system.^7^

To ensure CHW programs are effective, political will should be documented in community health policy and strategy.

Further, with these supportive policy frameworks in place, making the case for investment provides the evidence required to support resource mobilization, consequently reducing the funding gap. In South Africa, following the launch of the PHC re-engineering approach in 2012, which placed the ward-based outreach teams as an integral part of PHC, the National Department of Health commissioned the development of an investment case that provided the evidence to invest in the CHW program. All the interventions by the CHWs in maternal and child health, TB, HIV, hypertension, and diabetes demonstrated a decrease of 200,000 deaths and more than 4.8 million disability-adjusted life years averted over 10 years.^15^ Consequently, a saving of R30 billion (US$2 billion) could be made over the 10 years.^15^ Beyond the impact on health metrices, this investment case demonstrated a multiplier effect of 1.5 due to the additional salaries injected into the economy as well as additional gains of R143 billion (US$9.6 billion) to the gross domestic product from increased productivity due to the deaths averted over the 10 years. This evidence for investment facilitated decision making and resource mobilization; in 2017/2018, the PHC services in South Africa accounted for 30% of the consolidated national and provincial health budget.^13^

The Financing Alliance for Health continues to support countries in developing community health strategies and investment cases as a part of key efforts toward closing this CHW funding gap.

### 3. Ineffective and Fragmented Existing Donor Funding Structure

Whereas a gap in funding needs to be addressed, how existing funding for CHW program is utilized is ineffective. The funding flows are heavily fragmented with many donors funding vertical programs. The majority (60%) of the CHW program funding in sub-Saharan Africa is from donors and most of this is for vertical, disease-specific programs. Between 2007 and 2017, only 2.5% of the total health-related development assistance was for CHW programs, and most of the funding was for vertical, disease-control programs (HIV and other sexually transmitted infections, 38.9%; malaria, 19.8%; and reproductive health, 9.3%).^16^

Hsiao, a global expert in public health argued^17^:

*more money for health is a necessary but insufficient condition to better health. Money can be transformed into equitable, efficient, and effective health care only when appropriate financing methods are used …*

To overcome this fragmentation, national CHW programs must focus on designing integrated community health programs by ensuring that the programs are embedded within the PHC system as a linkage to the overall health care system, guiding the donors on the government priorities, and establishing mechanisms for accountability to encourage harmonization of donor funding.

To overcome fragmented funding of vertical programs, national CHW programs should integrate CHWs into the overall health care system, guide donors on government priorities, and establish accountability mechanisms.

**FIGURE 7:**
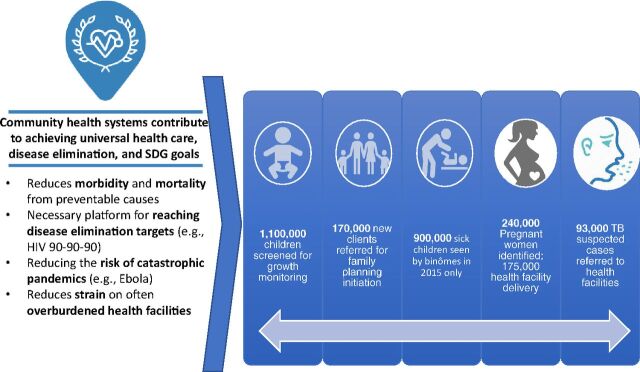
Health Impact of the Community Health Worker Program in Rwanda

Rwanda exemplifies this commitment of steering donors toward a unified vision. The government did not yield on their quest for channeling donor resources for horizontal PHC which contributed largely to their achievement of the health-related Millennium Development Goals before the target of 2015.^18^ Additionally, even though the Rwanda CHW program was heavily donor funded (87%), the government also allocated domestic resources (13% of total cost of CHW program) and set up other financing mechanisms, such as community-based health insurance and CHW cooperative societies, as a pathway toward sustainability (Figure 7).^13^

*A combination of community-based health insurance, community health workers, and good external partnerships led to the steepest reductions in child and maternal mortality ever recorded*. —Paul Kagame, President of the Republic of Rwanda

### 4. Suboptimal Impact of Community Health Worker Programs

**FIGURE 8:**
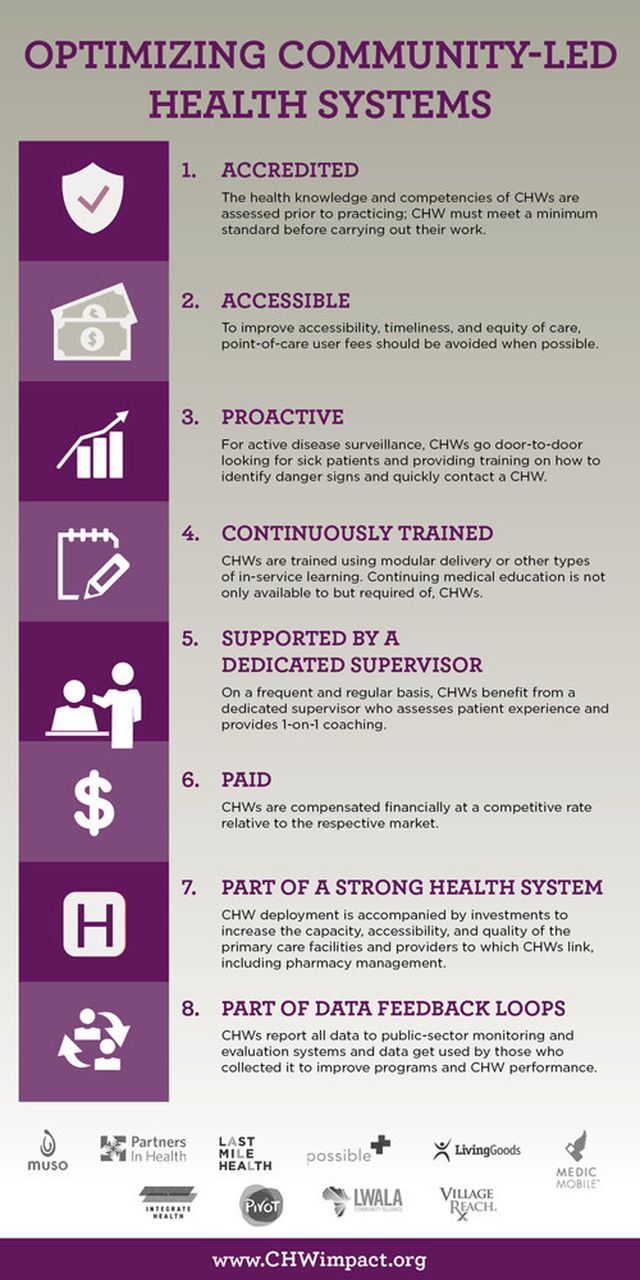
The 8 Design Principles to Design Optimized Community Programs

Unfortunately, recent evaluations of national CHW programs have revealed poor access, suboptimal quality in service delivery, and minimal impact on health indicators.^20^ This is because, beyond financing, CHWs programs must embrace a strong system design to be impactful. The patchy implementation that is common in SSA resulted in suboptimal impact.^14^ To ensure that CHW programs are effective, these 8 design principles and practices should be highly considered when designing national programs (Figure 8).^21^

## LESSONS LEARNED AND RECOMMENDATIONS

Over the past years, Financing Alliance for Health has learned several lessons in articulating the resource needs and advocating for closing the annual funding gap.

**FIGURE 9:**
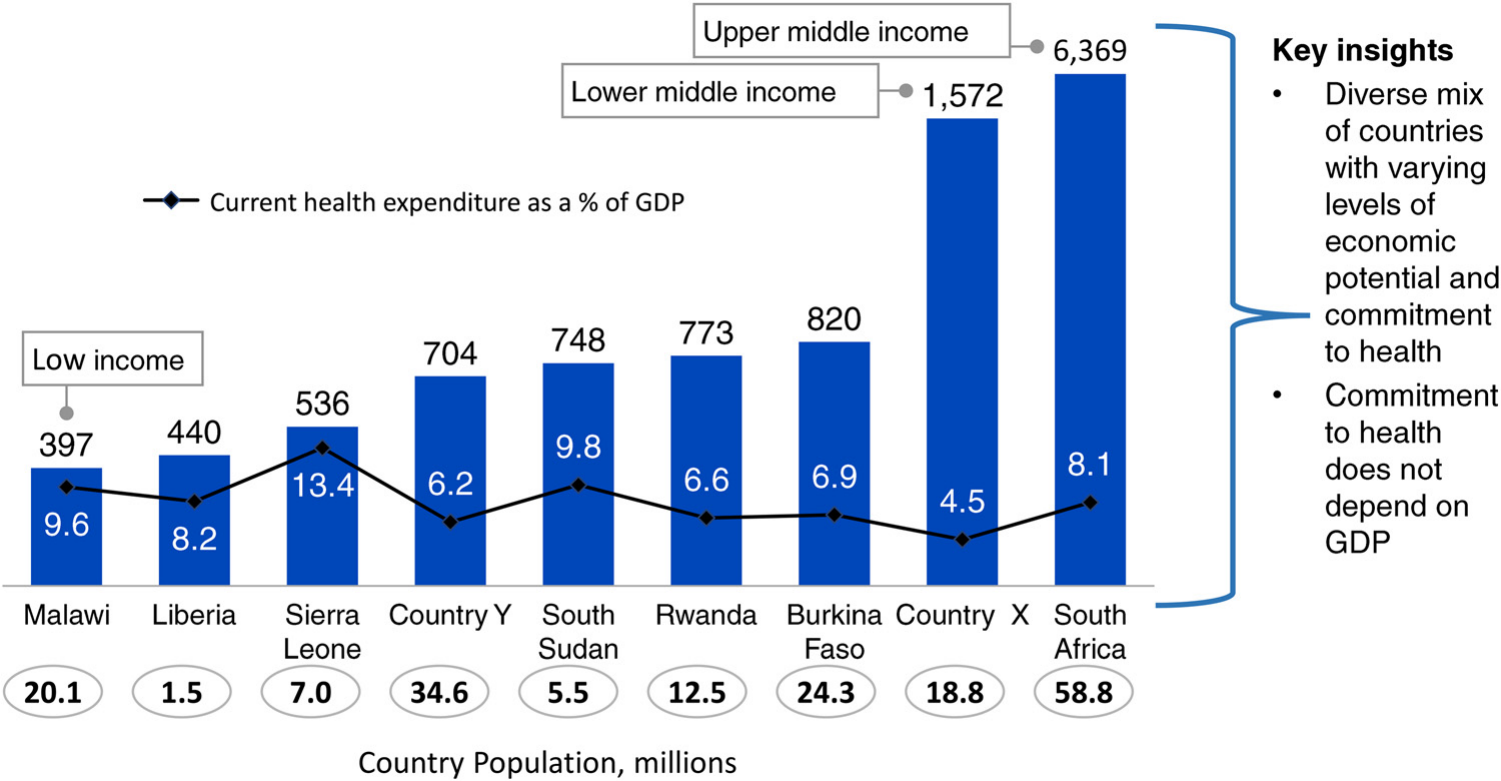
Correlation Between Country Income Status and Health Spending, Across 9 Countries in sub-Saharan Africa, Gross Domestic Product Per Capita, US$

- Prioritize political economy: It is not about the economic conditions prevailing in a country but rather the political prioritization of health that influences resource allocation. The socioeconomic status of a country does not necessarily correlate with health expenditure. Higher country income may not always translate to higher government spending on health, and low-income countries can build robust and ambitious, yet affordable, health systems despite their limited resources (unpublished data) (Figure 9).
- Establish supportive policies, strategies, and program design: Financing follows well-designed CHW programs that are embedded within PHC and are supported by policies and strategies to ensure integration of services and hence efficient and effective use of resources. We recommend that governments consider the 8 key design elements for effective CHW programs.
- Develop a strong case for investment: In the context of limited resources and competing health priorities, making the case for investment would facilitate advocating for more resources as well as guiding key decision makers on the allocation of the scarce resources. The Financing Alliance for Health has supported many governments in SSA in doing this and is open to supporting more countries in taking this critical step toward closing the community health funding gap.
- Governments and donors should strongly invest in integrated CHWs program over verticalized programs: Having one guiding vision and working collaboratively with donors toward an integrated CHW program will result in equitable, efficient, and effective use of existing funding. Donors should take a systems approach and focus their investments toward building resilient and sustainable platforms of delivery. These must respond to the breadth of disease burden of a country and be systems focused, a stark contrast to the disease-specific, “vertical” set-up.
- Tap into funding beyond the traditional sources: We must shift the global financing architecture to reflect and be responsive to realities on the ground. Current funding is insufficient and fragmented, leading to inefficiencies. In fact, despite a common narrative that community health and PHC are the most important investments for achieving universal health coverage, development assistance for CHW projects has been small, unstable, and declining over recent years. Increased funding is needed, starting with governments meeting their domestic health spending commitments, such as the Abuja declaration,^22^ which can in turn catalyze additional investments. There are massive opportunities to tap into capital markets and explore innovative approaches and instruments for health such as impact bonds, results-based financing, innovative private sector engagement, and blended financing. These efforts will help partner governments respond to the challenges of today, like COVID-19, and the communicable and noncommunicable disease challenges of the future.

## CONCLUSION

Concerted effort and renewed focus is direly needed to establish a resilient PHC platform that can reach every person with an integrated package of services. This system will need resources from multiple stakeholders to be efficient, equitable, and sustainable.

To those who have considered such a vision too expensive in the past, one needs only to look at what has been lost economically and socially in light of the pandemic crises such as Ebola and COVID-19. The question is no longer can we afford to build strong health systems that fundamentally address vulnerability for everyone, everywhere? The question is can we afford not to?

The question to ask is not can we afford to build strong health systems that fundamentally address vulnerability for everyone, everywhere? But rather, can we afford not to?

## Notes

**Peer Reviewed**
